# Systematic review and meta‐analysis on the impact of the levonorgestrel‐releasing intrauterine system in reducing risk of ovarian cancer

**DOI:** 10.1002/ijgo.13737

**Published:** 2021-06-08

**Authors:** Gloria D’Alessandro, Matteo Frigerio, Fabio Barra, Sergio Costantini, Claudio Gustavino, Simone Ferrero

**Affiliations:** ^1^ Academic Unit of Obstetrics and Gynecology IRCCS Ospedale Policlinico San Martino Genova Italy; ^2^ Department of Neurosciences, Rehabilitation, Ophthalmology, Genetics, Maternal and Child Health (DiNOGMI) University of Genova Genova Italy; ^3^ ASST Monza Ospedale San Gerardo Monza Italy; ^4^ Unit of Obstetrics and Gynecology IRCCS Ospedale Policlinico San Martino Genova Italy

**Keywords:** contraceptives cancer risk, intrauterine device, levonorgestrel‐releasing intrauterine system, ovarian cancer, risk factors for ovarian carcinoma

## Abstract

**Background:**

Ovarian carcinoma (OC) is one of the most widespread tumors in the world and is characterized by low survival rates.

**Objective:**

To determine whether the levonorgestrel‐releasing intrauterine system (LNG‐IUS) can prevent OC.

**Search strategy:**

The literature until December 2020 were systematically reviewed according to the PRISMA Statement for Reporting Systematic Reviews (PROSPERO: CRD42019137957).

**Selection criteria:**

Studies assessing the impact of LNG‐IUS on the risk of OC were included.

**Data collection and analysis:**

Data were extracted independently by two authors to ensure accuracy and consistency.

**Main results:**

A total of 34 323 records were obtained, of which three satisfied the inclusion criteria. In total, 1687 events of OC in a population of 20 461 311 person‐years were considered. Data pooling revealed that the use of LNG‐IUS did not confer a lower risk of OC relative to the never‐use of LNG‐IUS, with an estimated odds ratio of 0.66 (95% confidence interval 0.41–1.08; I^2 ^= 84%; *P *= 0.002).

**Conclusion:**

The meta‐analysis did not demonstrate a preventive role of LNG‐IUS on OC. However, it was carried out on a few papers, and a definitive conclusion on the topic still cannot be drawn. Further studies are indicated in the future to define the impact of LNG‐IUS on OC.

The meta‐analysis carried out on three papers did not demonstrate a preventive role of the levonorgestrel‐releasing intrauterine device on ovarian cancer.

## 1 INTRODUCTION

Ovarian carcinoma is one of the most widespread and lethal tumors in women: it represents the seventh most common cancer in the world and the eighth cause of death from cancer in women, with 3.6% of cases and 4.3% of deaths.^1^ The lifetime risk of developing ovarian tumors is estimated to be 1.4%. Ovarian carcinoma is usually asymptomatic at the early stages and tends to become symptomatic when ovarian carcinoma has already metastasized.^2^ After 5 years, the rates of survival change with stage at the time of diagnosis: 94% for localized disease; 73% for regional disease; and 28% for distant disease. The epithelial category represents 60% of all ovarian tumors, of which 90% are malignant.^2^ Sex cord‐stromal and germ cell tumors usually have benign features and are less frequent. The first serotype accounts for 8% of all ovarian tumors, afflicting postmenopausal women in particular, while the germ cell type accounts for 25% of all ovarian tumors, and it presents mostly at around 20 years of age.^3^

Given its heterogeneous entity, several theories have been postulated on the development of the disease. Serous tubal intraepithelial carcinoma in the fimbrias of the fallopian tubes is believed to be the major precursor of high‐grade serous cancer, while the surface epithelium of the ovary may give origin to low‐grade cancer.^4^ Endometrial cells or endometriosis play a role in the development of endometrioid and clear cell carcinoma,^5^ while gastrointestinal cells may be the precursors of mucinous ovarian carcinoma.^6^ Familiarity, reproductive history, and hormone fluctuations have been demonstrated to play a major role in the development of ovarian carcinoma. Nulliparity, postmenopausal hormone therapy (HT), and family history of ovarian carcinoma are directly related to the tumor risk.^7^, ^8^ Conversely, tubal ligation, pregnancies, and combined oral contraceptives (COC) have been inversely associated with the development of ovarian carcinoma. COCs are believed to prevent the disease by inhibiting ovulation, reducing menstrual bleeding, and retrograde menstruation.^9^ However, less evidence is available for progestin‐only hormonal contraceptives, in particular for levonorgestrel‐releasing intrauterine systems (LNG‐IUS).

LNG‐IUS was introduced in Norway in 1994, and its use is nowadays widespread.^10^ It was initially developed as a method of contraception and subsequently used also as therapy for heavy menstruation, dysmenorrhea, and endometrial protection.^11^ The device releases levonorgestrel (LNG) into the uterine cavity, where endometrial vessels soak up the hormone. The progestin level reaches a peak after the first few hours of insertion and a plateau after the first weeks^12^; thereafter, the systemic concentration decreases. The concentration of LNG becomes similar in the myometrium and the fallopian tubes, while locally, into the uterine cavity, the concentration is higher.^13^ While the impact of LNG‐IUS on endometrial epithelium is well defined and associated with a reduction in the risk of endometrial cancer, the effect on ovarian function is less clear. The first cycles may be anovulatory due to a higher concentration of hormone plasma. After the first year, the level of LNG decreases, and it becomes insufficient to suppress hypothalamic‐pituitary‐ovarian function without affecting systemic estradiol concentration.^14^, ^15^ Interestingly, recent studies suggest a protective role of LNG‐IUS not only on endometrial cancer but also on the development of ovarian carcinoma; however, its role is still unclear.^11^, ^16^, ^17^

As a consequence, the aim of the present review was to investigate the impact and effectiveness of the LNG‐IUS on risk of ovarian carcinoma by systematically reviewing the current literature and performing a meta‐analysis.

## 2 MATERIALS AND METHODS

The present systematic review was conducted and reported according to both the PRISMA Statement for Reporting Systematic Reviews and Meta‐Analyses^18^ and the Meta‐Analysis of Observational Studies in Epidemiology guidelines^19^ (PROSPERO registration code: CRD42019137957). Study objectives, eligibility criteria, outcome definitions, search strategy, process of data extraction, statistical analyses, and method of study quality assessment were all defined in a protocol. Investigators are experienced in systematic reviews.^20^, ^21^, ^22^, ^23^, ^24^

Studies assessing the impact of LNG‐IUS on the risk of ovarian carcinoma were included. Letters to the editor, conference abstracts, book chapters, guidelines, Cochrane reviews, and expert opinions were excluded. The occurrence of OC in the population exposed or not exposed to LNG‐IUS was considered an outcome measure.

To identify potentially eligible studies, PubMed, Scopus, Cochrane Library, and ISI Web of Science were searched (up to December 1, 2020) using EndNote x8 (Clarivate Analytics). A combination of keywords and text words were used: “levonorgestrel‐releasing”; “intrauterine system”; “intrauterine device”; “intrauterine implant”; “intrauterine contraceptives”; “ovarian cancer”; “ovarian carcinoma”; and “ovarian neoplasm.” An example of the complete search strategy used for the PubMed search is presented in Appendix S1. Two reviewers independently screened the titles and abstracts of the records that were retrieved through the database searches. No limitations to language were applied. A manual search was also performed using the reference lists of key articles to include additional relevant articles. Both reviewers independently recommended studies for the full‐text review. Full texts of records recommended by at least one reviewer were screened independently by the same two reviewers and assessed for inclusion in the systematic review. Disagreements between reviewers were solved by consensus.

Data were extracted using a piloted form specifically designed for capturing information on study and characteristics. Information about study design, settings, sample sizes and features of participants, exposure and outcome assessment, duration of follow‐up, confounding factors, and main findings were extracted from each study. The impact of the exposition was measured after the LNG‐IUS exposition, and the occurrence of ovarian carcinoma in the population was analyzed. Data were extracted independently by two authors to ensure accuracy and consistency. The authors were emailed about studies that were felt potentially might have unpublished data about the considered outcome. Two reviewers independently screened the full texts of records included in the systematic review and assessed the quality of the studies using the Newcastle‐Ottawa Scale (NOS). The NOS contains four items under the selection domain, one item under the comparability domain, and three items under the outcome domain. A star scoring system, from zero to nine stars, is used for the assessment of study quality, such that the highest‐quality studies are awarded one star per item, except for the comparability domain, for which two stars for a single item can be assigned. Disagreements between reviewers were solved by consensus.

The number of events of ovarian cancer in the population‐years according to the use (current or previous) versus non‐use of LNG IUS was collected from the considered studies. The pooling of results was done according to the random‐effects method of DerSimonian and Laird.^25^ The odds ratio (OR) was considered as the measure of effect. I^2^ and tau^2^ indexes were used to quantify heterogeneity between studies, and the null hypothesis that all studies share a common effect size was tested. All the analyses were performed using Revman 5.4 software.

## 3 RESULTS

**FIGURE 1:**
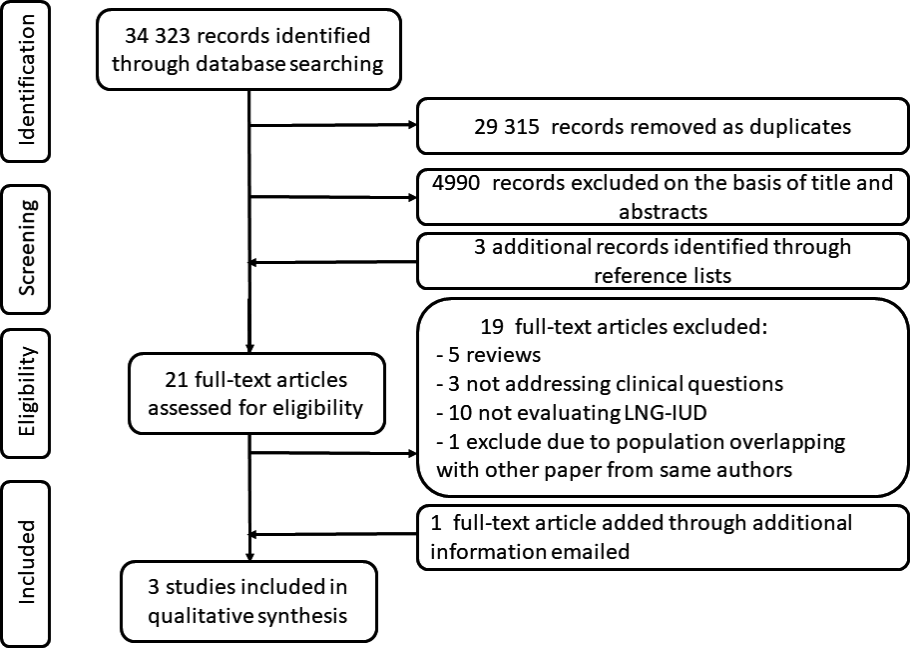
Search of electronic databases

The electronic database search provided a total of 34 323 results (Figure 1). After excluding any duplicates, there were 5 008 citations left. Of them, 4 990 were not relevant to the review based on title and abstract screening. Three further records found with a manual search were added. A total of 21 studies were considered for full‐text assessment, of which 19 were excluded for the following reasons: there were five reviews; three studies did not address clinical questions; 10 studies did not evaluate the LNG‐IUD; and one study had a population overlapping with an included study published by the same authors. None were excluded for languages other than English. Some answers were received from authors of potential includible papers, and an additional dataset was obtained.^26^ Finally, three studies met the inclusion criteria and were incorporated into the final assessment.^10^, ^26^, ^27^ The main characteristics of these studies are listed in Table 1. All considered studies showed satisfactory quality according to the NOS.

**TABLE 1:** Characteristics of studies dealing with the impact of LNG‐IUS on the risk of ovarian carcinoma^a^

First author	Year	Reference	Country	Study design	Users of LNG‐IUS (patients‐year)	OC in users of LNG‐IUS	Main results	Limitations	Newcastle‐Ottawa Scale		
									Selection	Comparability	Outcomes
Soini	2016	^27^	Finland	Prospective cohort study	1 083 126	77	SIR 0.59 (95% CI 0.47–0.73)	Not adjusted for use of oral contraceptives Lack of control population (comparison with expected events)	★★★	★★	★★★
Iversen	2018	^26^	Denmark	Prospective cohort study	708 111	44	Adjusted RR 0.72 (95% CI 0.53–0.99) for current or recent users of LNG‐IUS Adjusted RR 1.46 (95% CI 0.30–7.21) for users of LNG‐IUS with complete history of contraception Adjusted RR 0.84 (95% CI 0.53–1.35) for users of LNG‐IUS followed up to first switch in hormonal contraception	Lack of data on the type of hormonal contraception in former users (more than 1 year after discontinuing use)	★★★★	★	★★
Jareid	2018	^10^	Norway	Prospective cohort study	107 701	18	Multivariable‐adjusted RR 0.53 (95% CI 0.32–0.88)	Self‐reported exposure data Lack of adjustment for time since use of oral and other hormonal contraceptives	★	★★	★★★

Abbreviations: CI, confidence interval; LNG‐IUS, levonorgestrel‐releasing intrauterine system; OC, ovarian cancer; RR, relative risk; SIR, standardized incidence ratio.

^a^ Newcastle‐Ottawa Scale quality evaluation and variables considered.

Soini et al.^27^ led a prospective study cohort in Finland, evaluating the impact of the LNG‐IUS on ovarian and fallopian cancer in a population of women aged 30–49 years. After a long follow‐up period, with an average of 11.5 years (the maximum follow‐up was 20 years), they found 77 cases of ovarian carcinoma occurring in a cohort of 93 843 women who had been prescribed LNG‐IUS for menorrhagia, for a total of 1 083 126 person‐years. The authors performed an analysis of histological subtypes, observing a more significant decrease than expected in the occurrence of both invasive ovarian carcinoma (–41%; standardized incidence ratio [SIR] 0.59; 95% confidence interval [CI] 0.47–0.73) and borderline ovarian tumors (–24%; SIR 0.76; 95% CI 0.57–0.99). On the contrary, the risk of primary fallopian tube carcinoma was not affected (SIR 1.22; 95% CI 0.49–2.50) by LNG‐IUS. In particular, the authors reported the lowest risk for mucinous carcinoma (SIR 0.49; 95% CI 0.24–0.87) and the highest risk for serous type (SIR 0.75; 95% CI 0.55–0.99) compared to rates expected in the general population. Among other serotypes, the risk for endometrioid ovarian carcinoma was almost halved in users of the LNG‐IUS, while no significant conclusions could be made for ovarian clear cell carcinoma because of the low number of cases. Moreover, the incidence of mucinous, serous, or endometrioid ovarian carcinoma did not decrease after the first 5 years of follow‐up, but it remained stable. A considerable weakness of the study was that the observers did not adjust the results for any risk factors such as parity, tubal sterilization, hysterectomy, polycystic ovarian syndrome, family history of ovarian carcinoma, and use of COCs.

More recently, Iversen et al.^26^ evaluated the impact of hormonal contraception on ovarian carcinoma in a large population of premenopausal women in Denmark. The risk was stratified for different hormonal contraceptives, and the results were adjusted for calendar year, parity, age, education, tubal sterilization, hysterectomy, polycystic ovarian syndrome, endometriosis, and family history of breast and ovarian carcinoma. They collected data concerning duration, time since last use, and tumor histology, observing a decreased relative risk (RR) among current or recent users of any hormonal contraception (RR 0.66; 95% CI 0.58–0.76) compared to non‐users, with greater protection with longer durations of contraceptive use. They found that the protective effect diminished over time since the last use, and it was non‐significant by 10 years after stopping use. However, among users of progestogen‐only products, including the levonorgestrel housed in intrauterine systems, they did not observe any significant protective impact against ovarian carcinoma compared to combined COCs. Specifically, they collected the exposure to current and recent users of LNG‐IUS and restricted further the analysis to women with a complete history of exposure to contraception and women followed until the first switch in hormonal contraception. In the full cohort of current or recent users of LNG‐IUS, they observed a total adjusted RR of ovarian carcinoma of 0.72 (95% CI 0.53–0.99). However, when the analysis was restricted to women with a complete history of contraception exposure and women followed until the first switch in hormonal contraception, a protective effect was not demonstrated, with an adjusted RR of 1.46 (95% CI 0.30–7.21) and 0.84 (95% CI 0.53–1.35), respectively, compared to the non‐users. The authors concluded that, different from combined hormonal contraceptives, there is currently insufficient evidence to suggest similar protection among exclusive users of progestogen‐only products. A limitation was the lack of data on the specific type of hormonal contraception in the population of former users (more than 1 year after discontinuing use). However, the cohort of current and recent users of LNG‐IUS and never‐users resulted in a total of 1005 cases of ovarian carcinoma in a population of 16 989 624 person‐years.

The cohort study by Jareid et al.^10^ evaluated the risk of ovarian, endometrial, and breast carcinoma among a population of women of both premenopausal and postmenopausal age who used the intrauterine system, collecting data for analysis from the Norwegian Women and Cancer (NOWAC) Study. The median age at inclusion was 52 years, and the mean follow‐up time was 12.5 years, for a total of 1 305 435 person‐years. Among ever‐users of LNG‐IUS, there were 18 cases of epithelial ovarian cancer, 15 cases of endometrial cancer, and 297 cases of breast cancer. The risk of cancer was adjusted for use of COCs, age and menopausal status at the start of follow‐up, parity, level of physical activity and body mass index at enrollment, maternal history of breast cancer, and age at menarche. Whenever users were compared to never‐users of LNG‐IUS, the multivariable RR of ovarian, endometrial, and breast cancer was 0.53 (95% CI 0.32–0.88), 0.22 (0.13–0.40), and 1.03 (0.91–1.17), respectively. Limitations of the study included self‐reported exposure data and lack of adjustment for time since use of oral and other hormonal contraceptives that may have introduced confounding factors and/or risk of misclassification.

**FIGURE 2:**
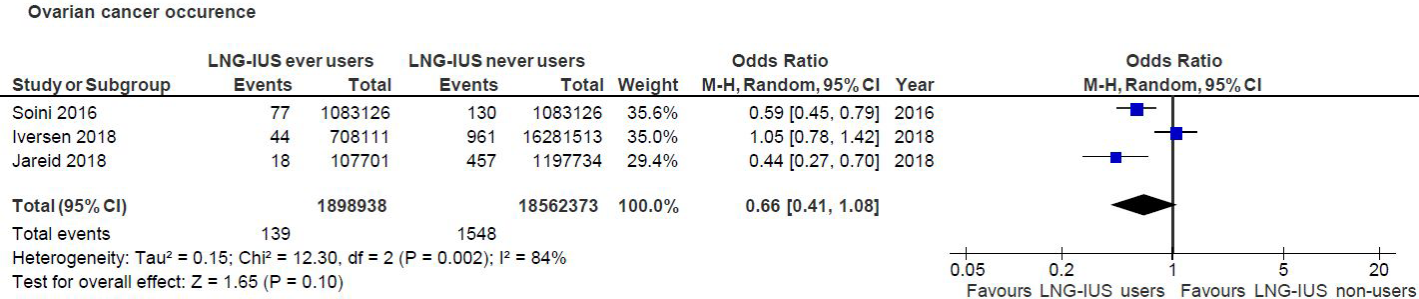
Forest plot. The risk of ovarian cancer among users of LNG‐IUS versus never‐users. Abbreviations: CI, confidence interval; LNG‐IUS, levonorgestrel intrauterine system

The considered studies were heterogeneous. Soini et al.^27^ compared the occurrence of ovarian carcinoma in the cohort of users of LNG‐IUS to the one expected in the general population, while the other two studies compared it with the actual case in the cohort of non‐user patients. Moreover, in the study by Iversen et al.,^26^ data on the specific type of hormonal contraception used were not available for the group of former users (more than 1 year after discontinuing use), and only data regarding current and recent users of LNG‐IUS were available for the aim of that meta‐analysis. In total, 1687 events of ovarian carcinoma in a population of 20 461 311 person‐years were considered. Data pooling revealed that the use of LNG‐IUS did not confer a lower risk of ovarian cancer relative to the never‐use of LNG‐IUS, with an estimated OR of 0.66 (95% CI 0.41–1.08; I^2 ^= 84%; *P *= 0.002) (Figure 2).

## 4 DISCUSSION

The aim of the present review was to investigate the impact of the LNG‐IUS on the risk of ovarian carcinoma by systematically reviewing the current literature. The meta‐analysis did not demonstrate a protective role of LNG‐IUS on the risk of ovarian carcinoma since an OR of 0.66 was found (95% CI 0.41–1.08) when users of LNG‐IUS were compared to never‐users of LNG‐IUS.

Concerning the impact of the duration of use of LNG‐IUS, only Jareid et al.^10^ reported the length of exposition in the analyzed population, reporting an exposition ranging from under 1 year to 14 years (with a median duration of 4 years). They observed 77% of cases of cancer occurring in women exposed for less than 7 years to LNG‐IUS. However, due to the small sample, the authors did not analyze the data, and the other included studies^26^, ^27^ did not specify the duration of the intrauterine device exposition; consequently, on the basis of the current evidence, it is not possible to establish if the duration of use provides benefits.

To date, the protective role of COCs towards ovarian cancer is well defined, and it is considered similar against main histotypes, including endometrioid, mucinous, and serous ovarian carcinoma.^26^ Suppression of ovulation seems to play a significant role, but the exact mechanisms by which COCs reduce the risk of ovarian cancer are still unclear. Interestingly, epidemiological data suggest that the use of COCs involves long‐lasting protection against ovarian cancer.^28^ On the contrary, there is insufficient evidence to suggest similar protection among exclusive users of progestogen‐only products, and this is even more uncertain concerning progestin‐releasing intrauterine devices. While immunohistochemical data indicate a protective effect of oral LNG on ovarian tissue in terms of proliferation index and karyometric abnormalities,^29^ evidence about the clinical impact on the prevention of ovarian carcinoma is conflicting.^30^, ^31^ Recently, a vast prospective nationwide cohort study reported no protective effect from progestogen‐only products on the risk of ovarian carcinoma.^26^

Similarly, the impact of intrauterine devices as a protective factor for ovarian carcinoma remains controversial. This issue had been already raised in 1989^32^ when a decreasing incidence of ovarian carcinoma (RR 0.8) in users of IUS was first reported. On the other hand, some years later, a prospective study cohort among premenopausal and postmenopausal women^33^ reported an adverse effect of IUS on the incidence of ovarian carcinoma (RR 1.76). Specifically, the role of non‐progestin intrauterine devices remains unclear, with meta‐analyses considering mixed populations including copper, stainless steel, and LNG‐releasing systems.^34^, ^35^ On the contrary, recent studies explicitly focusing on LNG‐IUS have suggested a protective role in the development of ovarian carcinoma,^10^, ^27^ but the impact of this family of devices is not well defined. Some authors proposed different mechanisms to explain the possible protective effect of the use of LNG‐IUS on the risk of ovarian carcinoma by intrauterine devices might alter the passage of carcinogenic agents towards the fimbriae by changing the intrauterine environment.^27^ Moreover, reduction in menstrual flow induced by levonorgestrel may be linked to less inflammatory changes in the fimbriae and reduced retrograde endometrial cell displacement, which are considered risk factors for ovarian carcinoma.^10^, ^27^, ^32^ Besides, the small amount of levonorgestrel systemically absorbed might bind to the ovarian epithelial cell receptors leading to protective molecular alterations.^27^ Lastly, the partial inhibition of ovulation provoked by the intrauterine system, especially during the first few months, may again play a protective role, similar to the one postulated for COCs.^36^

A previous meta‐analysis by Balayla et al.^34^ on two studies^10^, ^27^ reported a protective effect of LNG‐IUS on the occurrence of ovarian carcinoma, with a SIR estimate of 0.58 (95% CI 0.47–0.71). However, the considered studies carry some critical risk of bias. Soini et al.^27^ did not adjust the risk of ovarian carcinoma for the use of oral contraceptives, and rates of ovarian carcinoma are compared to those expected in the general population.^27^ Jareid et al.^10^ used self‐reported data about exposure to contraceptives and did not adjust the risk of ovarian carcinoma for time since use of oral and other hormonal contraceptives. Moreover, their meta‐analysis resulted in a limited population, involving 682 events of ovarian carcinoma in 3 471 687 person‐years.

The present meta‐analysis included a further study, which greatly increased the number of the considered population up to 1687 events of ovarian carcinoma in a population of 20 461 311 person‐years. This additional paper by Iversen et al.^26^ did not demonstrate a protective effect from exposure to LNG‐IUS among a large nationwide cohort of women, when restricted to women whose hormonal history was known and followed up to the first switch in hormonal contraception, accounting for the potential lingering effect of prior use of COCs.^26^ As a consequence, the data pooling revealed no effect of LNG‐IUS on risk of ovarian cancer. A limitation of the present meta‐analysis is the lack of stratification for histotypes since only one study investigated this parameter.^27^ Since endometrioid tumors originate from endometriotic cells^5^ and express high levels of progesterone receptors (PRs), it may be expected that LNG may involve a specific and more pronounced protective effect on this histotype.^37^ Another limitation can be found in the limited number of considered studies, revealing the urgent need for further studies on this topic.

The meta‐analysis could not demonstrate that LNG‐IUS has a preventive role in the development of ovarian cancer. However, since only a few papers were retrieved, a definitive conclusion on the topic cannot be drawn, and further studies are needed to better define the impact of LNG‐IUS on ovarian carcinoma. Moreover, the effect of the duration of exposure to LNG‐IUS and any beneficial role on cancer susceptibility while the device is inserted represent further interesting aspects to investigate in the future.

## CONFLICTS OF INTEREST

The authors have no conflicts of interest.

## AUTHOR CONTRIBUTIONS

GD was responsible for data collection and MF for data analysis and interpretation of results. GD and MF developed the project and wrote the manuscript. FB, SC, and CG contributed to critical revision, and SF was responsible for the conception of the study.

## Supporting information

Appendix S1

## References

[1] Ferlay J , Soerjomataram I , Dikshit R , et al. Cancer incidence and mortality worldwide: sources, methods and major patterns in GLOBOCAN 2012. Int J Cancer. 2015;136:E359‐E386. 25220842 10.1002/ijc.29210

[2] Zapata LB , Whiteman MK , Tepper NK , Jamieson DJ , Marchbanks PA , Curtis KM . Intrauterine device use among women with uterine fibroids: a systematic review. Contraception. 2010;82(1):41‐55. 20682142 10.1016/j.contraception.2010.02.011

[3] Chen VW , Ruiz B , Killeen JL , et al. Pathology and classification of ovarian tumors. Cancer. 2003;97(10 Suppl):2631‐2642. 12733128 10.1002/cncr.11345

[4] Crum CP , McKeon FD , Xian W . The oviduct and ovarian cancer: causality, clinical implications, and “targeted prevention”. Clin Obstet Gynecol. 2012;55(1):24‐35. 22343226 10.1097/GRF.0b013e31824b1725 PMC3319355

[5] Nezhat FR , Apostol R , Nezhat C , Pejovic T . New insights in the pathophysiology of ovarian cancer and implications for screening and prevention. Am J Obstet Gynecol. 2015;213(3):262‐267. 25818671 10.1016/j.ajog.2015.03.044

[6] Kelemen LE , Köbel M . Mucinous carcinomas of the ovary and colorectum: different organ, same dilemma. Lancet Oncol. 2011;12(11):1071‐1080. 21616717 10.1016/S1470-2045(11)70058-4

[7] Collaborative Group on Epidemiological Studies of Ovarian Cancer , Beral V , Gaitskell K , et al. Menopausal hormone use and ovarian cancer risk: individual participant meta‐analysis of 52 epidemiological studies. Lancet. 2015;385(9980):1835‐1842. 25684585 10.1016/S0140-6736(14)61687-1 PMC4427760

[8] Modugno F , Laskey R , Smith AL , Andersen CL , Haluska P , Oesterreich S . Hormone response in ovarian cancer: time to reconsider as a clinical target? Endocr Relat Cancer. 2012;19(6):R255‐R279. 23045324 10.1530/ERC-12-0175 PMC3696394

[9] Cramer DW , Xu H . Epidemiologic evidence for uterine growth factors in the pathogenesis of ovarian cancer. Ann Epidemiol. 1995;5(4):310‐314. 8520714 10.1016/1047-2797(94)00098-e

[10] Jareid M , Thalabard JC , Aarflot M , Bøvelstad HM , Lund E , Braaten T . Levonorgestrel‐releasing intrauterine system use is associated with a decreased risk of ovarian and endometrial cancer, without increased risk of breast cancer. Results from the NOWAC Study. Gynecol Oncol. 2018;149(1):127‐132. 29482839 10.1016/j.ygyno.2018.02.006

[11] Soini T , Hurskainen R , Grénman S , Mäenpää J , Paavonen J , Pukkala E . Cancer risk in women using the levonorgestrel‐releasing intrauterine system in Finland. Obstet Gynecol. 2014;124(2 Pt 1):292‐299. 25004338 10.1097/AOG.0000000000000356

[12] Luukkainen T , Lähteenmäki P , Toivonen J . Levonorgestrel‐releasing intrauterine device. Ann Med. 1990;22(2):85‐90. 10.3109/07853899009147248 2113816

[13] Nilsson CG , Haukkamaa M , Vierola H , Luukkainen T . Tissue concentrations of levonorgestrel in women using a levonorgestrel‐releasing IUD. Clin Endocrinol (Oxf). 1982;17(6):529‐536. 6819901 10.1111/j.1365-2265.1982.tb01625.x

[14] Nilsson CG , Lähteenmäki PLA , Luukkainen T . Ovarian function in amenorrheic and menstruating users of a levonorgestrel‐releasing intrauterine device. Fertil Steril. 1984;41(1):52‐55. 6420203

[15] Xiao B , Zhou L , Zhang X , Luukkainen T , Allonen H . Pharmacokinetic and pharmacodynamic studies of levonorgestrel‐releasing intrauterine device. Contraception. 1990;41(4):353‐362. 2335100 10.1016/0010-7824(90)90035-t

[16] Heikkinen S , Koskenvuo M , Malila N , Sarkeala T , Pukkala E , Pitkäniemi J . Use of exogenous hormones and the risk of breast cancer: results from self‐reported survey data with validity assessment. Cancer Causes Control. 2016;27(2):249‐258. 26667320 10.1007/s10552-015-0702-5

[17] Lyytinen HK , Dyba T , Ylikorkala O , Pukkala EI . A case‐control study on hormone therapy as a risk factor for breast cancer in Finland: intrauterine system carries a risk as well. Int J Cancer. 2010;126(2):483‐489. 19588504 10.1002/ijc.24738

[18] Liberati A , Altman DG , Tetzlaff J , et al. The PRISMA statement for reporting systematic reviews and meta‐analyses of studies that evaluate health care interventions: explanation and elaboration. PLoS Med. 2009;6(7):e1000100. 19621070 10.1371/journal.pmed.1000100 PMC2707010

[19] Stroup DF , Berlin JA , Morton SC , et al. Meta‐analysis of observational studies in epidemiology: a proposal for reporting. Meta‐analysis of Observational Studies in Epidemiology (MOOSE) group. JAMA. 2000;283(15):2008‐2012. 10789670 10.1001/jama.283.15.2008

[20] Barba M , Schivardi G , Manodoro S , Frigerio M . Obstetric outcomes after uterus‐sparing surgery for uterine prolapse: a systematic review and meta‐analysis. Eur J Obstet Gynecol Reprod Biol. 2021;256:333‐338. 33271407 10.1016/j.ejogrb.2020.11.054

[21] De Vitis LA , Barba M , Lazzarin S , et al. Female genital hair‐thread tourniquet syndrome: a case report and literature systematic review. J Pediatr Adolesc Gynecol. 2020;S1083‐3188(20)30283‐7. 10.1016/j.jpag.2020.07.007 32693024

[22] Frigerio M , Mastrolia SA , Spelzini F , Manodoro S , Yohay D , Weintraub AY . Long‐term effects of episiotomy on urinary incontinence and pelvic organ prolapse: a systematic review. Arch Gynecol Obstet. 2019;299(2):317‐325. 30564925 10.1007/s00404-018-5009-9

[23] Bogani G , Borghi C , Leone Roberti Maggiore U , et al. Minimally invasive surgical staging in early‐stage ovarian carcinoma: a systematic review and meta‐analysis. J Minim Invasive Gynecol. 2017;24(4):552‐562. 28223182 10.1016/j.jmig.2017.02.013

[24] Scala C , Morlando M , Familiari A , et al. Fetal tricuspid regurgitation in the first trimester as a screening marker for congenital heart defects: systematic review and meta‐analysis. Fetal Diagn Ther. 2017;42(1):1‐8. 28482343 10.1159/000455947

[25] DerSimonian R , Laird N . Meta‐analysis in clinical trials. Control Clin Trials. 1986;7(3):177‐188. 3802833 10.1016/0197-2456(86)90046-2

[26] Iversen L , Fielding S , Lidegaard Ø , Mørch LS , Skovlund CW , Hannaford PC . Association between contemporary hormonal contraception and ovarian cancer in women of reproductive age in Denmark: prospective, nationwide cohort study. BMJ. 2018;362:k3609. 30257920 10.1136/bmj.k3609 PMC6283376

[27] Soini T , Hurskainen R , Grénman S , Mäenpää J , Paavonen J , Pukkala E . Impact of levonorgestrel‐releasing intrauterine system use on the cancer risk of the ovary and fallopian tube. Acta Oncol (Madr). 2016;55(11):1281‐1284. 10.1080/0284186X.2016.1175660 27148621

[28] Collaborative Group on Epidemiological Studies of Ovarian Cancer , Beral V , Doll R , Hermon C , Peto R , Reeves G . Ovarian cancer and oral contraceptives: collaborative reanalysis of data from 45 epidemiological studies including 23,257 women with ovarian cancer and 87,303 controls. Lancet. 2008;371(9609):303‐314. 18294997 10.1016/S0140-6736(08)60167-1

[29] Dunselman GAJ , Vermeulen N , Becker C , et al. ESHRE guideline: management of women with endometriosis. Hum Reprod. 2014;29(3):400‐412. 24435778 10.1093/humrep/det457

[30] Kumle M , Weiderpass E , Braaten T , Adami HO , Lund E . Risk for invasive and borderline epithelial ovarian neoplasias following use of hormonal contraceptives: The Norwegian‐Swedish Women’s Lifestyle and Health Cohort Study. Br J Cancer. 2004;90(7):1386‐1391. 15054460 10.1038/sj.bjc.6601715 PMC2409682

[31] Koskela‐Niska V , Pukkala E , Lyytinen H , Ylikorkala O , Dyba T . Effect of various forms of postmenopausal hormone therapy on the risk of ovarian cancer ‐ a population‐based case control study from Finland. Int J Cancer. 2013;133(7):1680‐1688. 23526244 10.1002/ijc.28167

[32] Booth M , Beral V , Smith P . Risk factors for ovarian cancer: a case‐control study. Br J Cancer. 1989;60(4):592‐598. 2679848 10.1038/bjc.1989.320 PMC2247100

[33] Perets R , Drapkin R . It’s totally tubular…Riding the new wave of ovarian cancer research. Cancer Res. 2016;76(1):10‐17. 26669862 10.1158/0008-5472.CAN-15-1382 PMC4703449

[34] Balayla J , Gil Y , Lasry A , Mitric C . Ever‐use of the intra‐uterine device and the risk of ovarian cancer. J Obstet Gynaecol. 2020;1–6. 10.1080/01443615.2020.1789960 33045859

[35] Wheeler LJ , Desanto K , Teal SB , Sheeder J , Guntupalli SR . Intrauterine device use and ovarian cancer risk: a systematic review and meta‐analysis. Obstet Gynecol. 2019;134(4):791‐800. 31503144 10.1097/AOG.0000000000003463

[36] Rodriguez GC , Walmer DK , Cline M , et al. Effect of progestin on the ovarian epithelium of macaques: cancer prevention through apoptosis? J Soc Gynecol Investig. 1998;5(5):271‐276. 10.1016/s1071-5576(98)00017-3 9773403

[37] Ness RB , Dodge RC , Edwards RP , Baker JA , Moysich KB . Contraception methods, beyond oral contraceptives and tubal ligation, and risk of ovarian cancer. Ann Epidemiol. 2011;21(3):188‐196. 21109450 10.1016/j.annepidem.2010.10.002 PMC3052991

