# Supplementary material for: Systematic review and meta‐analysis on the impact of the levonorgestrel‐releasing intrauterine system in reducing risk of ovarian cancer
Source: Int J Gynaecol Obstet. 2021 Jun 8;156(3):418–24. doi: 10.1002/ijgo.13737 (PMC9290617; doi:10.1002/ijgo.13737)
Supplement: Supplementary file 1 — Appendix S1 [file IJGO-156-418-s001.docx]

**Supplementary Table 1**. Search strategy.

|  | **levonorgestrel-releasing, ovarian cancer 38** |
| --- | --- |
| [#1](http://www.ncbi.nlm.nih.gov/pubmed/advanced) | "levonorgestrel-releasing"[All Fields] AND ("ovarian neoplasms"[MeSH Terms] OR ("ovarian"[All Fields] AND "neoplasms"[All Fields]) OR "ovarian neoplasms"[All Fields] OR ("ovarian"[All Fields] AND "cancer"[All Fields]) OR "ovarian cancer"[All Fields]) |
|  | **levonorgestrel-releasing, ovarian carcinoma 33** |
| #2 | "levonorgestrel-releasing"[All Fields] AND ("ovarian neoplasms"[MeSH Terms] OR ("ovarian"[All Fields] AND "neoplasms"[All Fields]) OR "ovarian neoplasms"[All Fields] OR ("ovarian"[All Fields] AND "carcinoma"[All Fields]) OR "ovarian carcinoma"[All Fields]) |
|  | **levonorgestrel-releasing, ovarian neoplasm 34** |
| #3 | "levonorgestrel-releasing"[All Fields] AND ("ovarian neoplasms"[MeSH Terms] OR ("ovarian"[All Fields] AND "neoplasms"[All Fields]) OR "ovarian neoplasms"[All Fields] OR ("ovarian"[All Fields] AND "neoplasm"[All Fields]) OR "ovarian neoplasm"[All Fields]) |
|  | **intrauterine system, ovarian cancer 82** |
| #4 | ("intrauterin"[All Fields] OR "intrauterine"[All Fields]) AND ("drug delivery systems"[MeSH Terms] OR ("drug"[All Fields] AND "delivery"[All Fields] AND "systems"[All Fields]) OR "drug delivery systems"[All Fields] OR "system"[All Fields] OR "system s"[All Fields] OR "systems"[All Fields]) AND ("ovarian neoplasms"[MeSH Terms] OR ("ovarian"[All Fields] AND "neoplasms"[All Fields]) OR "ovarian neoplasms"[All Fields] OR ("ovarian"[All Fields] AND "cancer"[All Fields]) OR "ovarian cancer"[All Fields]) |
|  | **intrauterine system, ovarian carcinoma 61** |
| #5 | ("intrauterin"[All Fields] OR "intrauterine"[All Fields]) AND ("drug delivery systems"[MeSH Terms] OR ("drug"[All Fields] AND "delivery"[All Fields] AND "systems"[All Fields]) OR "drug delivery systems"[All Fields] OR "system"[All Fields] OR "system s"[All Fields] OR "systems"[All Fields]) AND ("ovarian neoplasms"[MeSH Terms] OR ("ovarian"[All Fields] AND "neoplasms"[All Fields]) OR "ovarian neoplasms"[All Fields] OR ("ovarian"[All Fields] AND "carcinoma"[All Fields]) OR "ovarian carcinoma"[All Fields]) |
|  | **intrauterine system, ovarian neoplasm 62** |
| #6 | ("intrauterin"[All Fields] OR "intrauterine"[All Fields]) AND ("drug delivery systems"[MeSH Terms] OR ("drug"[All Fields] AND "delivery"[All Fields] AND "systems"[All Fields]) OR "drug delivery systems"[All Fields] OR "system"[All Fields] OR "system s"[All Fields] OR "systems"[All Fields]) AND ("ovarian neoplasms"[MeSH Terms] OR ("ovarian"[All Fields] AND "neoplasms"[All Fields]) OR "ovarian neoplasms"[All Fields] OR ("ovarian"[All Fields] AND "neoplasm"[All Fields]) OR "ovarian neoplasm"[All Fields]) |
|  | **intrauterine device, ovarian cancer 209** |
| #7 | ("intrauterine devices"[MeSH Terms] OR ("intrauterine"[All Fields] AND "devices"[All Fields]) OR "intrauterine devices"[All Fields] OR ("intrauterine"[All Fields] AND "device"[All Fields]) OR "intrauterine device"[All Fields]) AND ("ovarian neoplasms"[MeSH Terms] OR ("ovarian"[All Fields] AND "neoplasms"[All Fields]) OR "ovarian neoplasms"[All Fields] OR ("ovarian"[All Fields] AND "cancer"[All Fields]) OR "ovarian cancer"[All Fields]) |
|  | **intrauterine device, ovarian carcinoma 160** |
| #8 | ("intrauterine devices"[MeSH Terms] OR ("intrauterine"[All Fields] AND "devices"[All Fields]) OR "intrauterine devices"[All Fields] OR ("intrauterine"[All Fields] AND "device"[All Fields]) OR "intrauterine device"[All Fields]) AND ("ovarian neoplasms"[MeSH Terms] OR ("ovarian"[All Fields] AND "neoplasms"[All Fields]) OR "ovarian neoplasms"[All Fields] OR ("ovarian"[All Fields] AND "carcinoma"[All Fields]) OR "ovarian carcinoma"[All Fields]) |
|  | **intrauterine device, ovarian neoplasm 159** |
| #9 | ("intrauterine devices"[MeSH Terms] OR ("intrauterine"[All Fields] AND "devices"[All Fields]) OR "intrauterine devices"[All Fields] OR ("intrauterine"[All Fields] AND "device"[All Fields]) OR "intrauterine device"[All Fields]) AND ("ovarian neoplasms"[MeSH Terms] OR ("ovarian"[All Fields] AND "neoplasms"[All Fields]) OR "ovarian neoplasms"[All Fields] OR ("ovarian"[All Fields] AND "neoplasm"[All Fields]) OR "ovarian neoplasm"[All Fields]) |
|  | **intrauterine implant, ovarian cancer 33** |
| #10 | ("intrauterin"[All Fields] OR "intrauterine"[All Fields]) AND ("embryo implantation"[MeSH Terms] OR ("embryo"[All Fields] AND "implantation"[All Fields]) OR "embryo implantation"[All Fields] OR "implantation"[All Fields] OR "implant"[All Fields] OR "implant s"[All Fields] OR "implantability"[All Fields] OR "implantable"[All Fields] OR "implantables"[All Fields] OR "implantate"[All Fields] OR "implantated"[All Fields] OR "implantates"[All Fields] OR "implantations"[All Fields] OR "implanted"[All Fields] OR "implanter"[All Fields] OR "implanters"[All Fields] OR "implanting"[All Fields] OR "implantion"[All Fields] OR "implantitis"[All Fields] OR "implants"[All Fields]) AND ("ovarian neoplasms"[MeSH Terms] OR ("ovarian"[All Fields] AND "neoplasms"[All Fields]) OR "ovarian neoplasms"[All Fields] OR ("ovarian"[All Fields] AND "cancer"[All Fields]) OR "ovarian cancer"[All Fields]) |
|  | **intrauterine implant, ovarian carcinoma 20** |
| #11 | ("intrauterin"[All Fields] OR "intrauterine"[All Fields]) AND ("embryo implantation"[MeSH Terms] OR ("embryo"[All Fields] AND "implantation"[All Fields]) OR "embryo implantation"[All Fields] OR "implantation"[All Fields] OR "implant"[All Fields] OR "implant s"[All Fields] OR "implantability"[All Fields] OR "implantable"[All Fields] OR "implantables"[All Fields] OR "implantate"[All Fields] OR "implantated"[All Fields] OR "implantates"[All Fields] OR "implantations"[All Fields] OR "implanted"[All Fields] OR "implanter"[All Fields] OR "implanters"[All Fields] OR "implanting"[All Fields] OR "implantion"[All Fields] OR "implantitis"[All Fields] OR "implants"[All Fields]) AND ("ovarian neoplasms"[MeSH Terms] OR ("ovarian"[All Fields] AND "neoplasms"[All Fields]) OR "ovarian neoplasms"[All Fields] OR ("ovarian"[All Fields] AND "carcinoma"[All Fields]) OR "ovarian carcinoma"[All Fields]) |
|  | **intrauterine implant, ovarian neoplasm 19** |
| #12 | ("intrauterin"[All Fields] OR "intrauterine"[All Fields]) AND ("embryo implantation"[MeSH Terms] OR ("embryo"[All Fields] AND "implantation"[All Fields]) OR "embryo implantation"[All Fields] OR "implantation"[All Fields] OR "implant"[All Fields] OR "implant s"[All Fields] OR "implantability"[All Fields] OR "implantable"[All Fields] OR "implantables"[All Fields] OR "implantate"[All Fields] OR "implantated"[All Fields] OR "implantates"[All Fields] OR "implantations"[All Fields] OR "implanted"[All Fields] OR "implanter"[All Fields] OR "implanters"[All Fields] OR "implanting"[All Fields] OR "implantion"[All Fields] OR "implantitis"[All Fields] OR "implants"[All Fields]) AND ("ovarian neoplasms"[MeSH Terms] OR ("ovarian"[All Fields] AND "neoplasms"[All Fields]) OR "ovarian neoplasms"[All Fields] OR ("ovarian"[All Fields] AND "neoplasm"[All Fields]) OR "ovarian neoplasm"[All Fields]) |
|  | **intrauterine contraceptives, ovarian cancer 203** |
| #13 | ("intrauterin"[All Fields] OR "intrauterine"[All Fields]) AND ("contracept"[All Fields] OR "contracepted"[All Fields] OR "contracepting"[All Fields] OR "contraception"[MeSH Terms] OR "contraception"[All Fields] OR "contraceptions"[All Fields] OR "contraceptive agents"[Pharmacological Action] OR "contraceptive agents"[MeSH Terms] OR ("contraceptive"[All Fields] AND "agents"[All Fields]) OR "contraceptive agents"[All Fields] OR "contraceptives"[All Fields] OR "contraceptive devices"[MeSH Terms] OR ("contraceptive"[All Fields] AND "devices"[All Fields]) OR "contraceptive devices"[All Fields] OR "contraceptive"[All Fields] OR "contraceptive s"[All Fields] OR "contraceptively"[All Fields]) AND ("ovarian neoplasms"[MeSH Terms] OR ("ovarian"[All Fields] AND "neoplasms"[All Fields]) OR "ovarian neoplasms"[All Fields] OR ("ovarian"[All Fields] AND "cancer"[All Fields]) OR "ovarian cancer"[All Fields]) |
|  | **intrauterine contraceptives, ovarian carcinoma 160** |
| #14 | ("intrauterin"[All Fields] OR "intrauterine"[All Fields]) AND ("contracept"[All Fields] OR "contracepted"[All Fields] OR "contracepting"[All Fields] OR "contraception"[MeSH Terms] OR "contraception"[All Fields] OR "contraceptions"[All Fields] OR "contraceptive agents"[Pharmacological Action] OR "contraceptive agents"[MeSH Terms] OR ("contraceptive"[All Fields] AND "agents"[All Fields]) OR "contraceptive agents"[All Fields] OR "contraceptives"[All Fields] OR "contraceptive devices"[MeSH Terms] OR ("contraceptive"[All Fields] AND "devices"[All Fields]) OR "contraceptive devices"[All Fields] OR "contraceptive"[All Fields] OR "contraceptive s"[All Fields] OR "contraceptively"[All Fields]) AND ("ovarian neoplasms"[MeSH Terms] OR ("ovarian"[All Fields] AND "neoplasms"[All Fields]) OR "ovarian neoplasms"[All Fields] OR ("ovarian"[All Fields] AND "carcinoma"[All Fields]) OR "ovarian carcinoma"[All Fields]) |
|  | **intrauterine contraceptives+ovarian neoplasm 160** |
| #15 | ("intrauterin"[All Fields] OR "intrauterine"[All Fields]) AND ("contracept"[All Fields] OR "contracepted"[All Fields] OR "contracepting"[All Fields] OR "contraception"[MeSH Terms] OR "contraception"[All Fields] OR "contraceptions"[All Fields] OR "contraceptive agents"[Pharmacological Action] OR "contraceptive agents"[MeSH Terms] OR ("contraceptive"[All Fields] AND "agents"[All Fields]) OR "contraceptive agents"[All Fields] OR "contraceptives"[All Fields] OR "contraceptive devices"[MeSH Terms] OR ("contraceptive"[All Fields] AND "devices"[All Fields]) OR "contraceptive devices"[All Fields] OR "contraceptive"[All Fields] OR "contraceptive s"[All Fields] OR "contraceptively"[All Fields]) AND ("ovarian neoplasms"[MeSH Terms] OR ("ovarian"[All Fields] AND "neoplasms"[All Fields]) OR "ovarian neoplasms"[All Fields] OR ("ovarian"[All Fields] AND "neoplasm"[All Fields]) OR "ovarian neoplasm"[All Fields]) |
